# Virus is a Signal for the Host Cell

**DOI:** 10.1007/s12304-015-9245-0

**Published:** 2015-07-17

**Authors:** Jordi Gómez, Ascensión Ariza-Mateos, Isabel Cacho

**Affiliations:** Laboratory of RNA Archeology, Instituto de Parasitología y Biomedicina ‘López-Neyra’, Consejo Superior de Ivestigaciones Científicas, Armilla 18100 Granada, Spain; Centro de Investigación Biomedicina En Red de enfermedades hepáticas y digestivas, Barcelona, Spain

**Keywords:** Molecular struggle, tRNA-like, IRES, Roux, Nietzsche, Eigen

## Abstract

Currently, the concept of the cell as a society or an ecosystem of molecular elements is gaining increasing acceptance. The basic idea arose in the 19th century, from the surmise that there is not just a single unit underlying an individual’s appearance, but a plurality of entities with both collaborative and conflicting relationships. The following hypothesis is based around this model. The incompatible activities taking place between different original elements, which were subsumed into the first cell and could not be eliminated, had to be controlled very closely. Similarly, a strong level of control had to be developed over many cellular elements after the cell changed its genome to DNA. We assume that at least some of those original RNA agents and other biomolecules which carry incompatibilities and risks, are retained within current cells, although they are now under strict control. A virus functions as a signal informing these repressed cellular RNAs and other elements of ancient origin how to restore suppressed degrees of molecular freedom, favoring pre-existing molecular affinities and activities, re-establishing ancient molecular webs of interactions, and giving fragments of ancient coded information (mostly in the form of RNA structural motifs) the opportunity to be re-expressed. Collectively, these newly activated mechanisms lead to different possibilities for pathological cell states. All these processes are opposed by cell-control mechanisms. Thus, in this new scenario, the battle is considered intracellular rather than between the virus and the cell. And so the virus is treated as the signal that precipitates the cell’s change from a latent to an active pathological state.

## Introduction

The unitary nature of the cell was criticized from a mechanistic and Darwinian perspective by one of the great scientists of the nineteenth century, Wilhelm Roux, in his 1881 book *The Struggle of the Parts in the Organism* (*Der Kampf der Teile im Organismus*) (Roux 2012). In this work, Roux proposed that the struggle must not only take place between organisms of the same species, but also on other, lower levels, such as the cellular or molecular levels. Examples at the cellular level could include the outgrowth of a cellular clone expressing a specific antibody after an antigen presentation or the selection of subsets of neuronal webs during development (Heams 2012). At the subcellular level these include the sequestered functional chloroplasts that can still be found in sea slugs which use them as a source of stored food reserves for sustenance during starvation periods (Christa et al. 2014a, b). The “molecular struggle” has also been related to several different forms of “intragenomic conflicts”, where genes do not follow the same laws of transmission (Heams 2012; Crespi and Nosil 2013); in the hypothetical genome reduction occurring during organelle (Bapteste and Gribaldo 2003) or other endosymbiosis (Husnik et al. 2013); or, possibly, for potential antagonist interaction between biomolecules within the same cell, for example in addiction modules in bacteria (Villarreal and Witzany 2013; Villarreal 2009).

Roux’s idea was taken up by Nietzsche, who integrated it into his metaphysical circuits: “Although the “units” are required to tell the tale, this doesn’t mean that such units “exist”. The concept of the unity is derived from our concept of “I”, which is our oldest article of faith. If we never considered ourselves as units, we would never have reached the concept of “thing”. Maybe we have been warned too late that our conception of “I” does not guarantee anything that can be referred to as a real unit” (Nietzsche 1968, Aphorism 520). Nietzsche suggest an ontology for interpreting organisms as a temporary result of a never-ending conflict, either inner or with the external world (Tauber 1994; Stiegler 2001).”Continuous becoming does not allow us to speak of individuals, etc.: the number of beings varies constantly…” (Nietzsche 1968, Aphorism 628). For Nietzsche, the most frequent changes would result from integrating the new and adapting it to what already existed (compressively reviewed in Stiegler 2001). Science has not yet provided a theoretical formulation capable of meeting the experimental exploration challenge of the Nietzschean view of his “being”: a metamorphic and active subject in directing its own becoming in contraposition to the passive Darwinian “unit of selection”. Nevertheless at present something is changing, new theoretical expressions oppose classical hierarchical ontology and, active and “far-seen” agents are being seriously considered (Eigen and Biebricher 1988; Gómez and Cacho 2001; Villarreal 2009; Bapteste and Dupre 2013; Sharov 2014; Noble 2015).

In general, viral infection is considered in terms of a relationship between two elements: the virus and host cell, or virus and the ribosome (Forterre and Prangishvili 2009). However, there might be problems for these clear splits as experts recognize the fact that the origin of the cell’s constitution is still a great enigma in biology (Koonin 2014). Moreover, since viruses were first discovered, experts have still not reached agreement as to whether they are by-products of cell evolution or living organisms that pre-date cells (Moreira and López-García 2009; Villarreal and Witzany 2010). In our opinion, new data from virology also disrupts the theory of the cell as a coherent unit which tries to defend itself against infection: recently, it has been demonstrated that among genes induced by interferon (the paradigm of antiviral defense) nearly a third of them activate rather than suppress infection in a large set of (+) single-strand RNA viruses (Schoggins et al. 2014).

## The Hypothesis

### Unity of Patholological and Healthy States

Here, we propose a conceptual inversion. We begin by accepting the fact that the cell contains a multitude of factors and activities integrated into its subcellular groupings and molecular networks which are repressed. These factors (if expressed and coordinated) may cause genomic or cytoplasmic alterations, and then, the cell would acquire a range of distinct pathological states. That is to say, we accept that within the cell there are multiple grave dangers, and a healthy cell includes multiple potential pathologies. We will refer to this healthy condition as the latent pathological state of the cell. Necessarily, it may be argued that the cell is derived from a pathology-repressing molecular organization: the cell would not have existed if numerous incompatible activities of the original RNAs and other biomolecules were capable of avoiding diffusion, being under hierarchical organization, compartmentalization, being thermodynamically disadvantaged, kinetically slowed, time-synchronized, covalently modified, and so on. This surmise is necessary when the overall objective is to permit only the expression of the compatible qualities and coherent signals and codes (Barbieri 2008), i.e., those which foster collaboration between the different molecules that were finally enclosed within the cell. There may have been a new, significant wave of repression during the move from the RNA world to DNA genomes, in which, for example, many RNA activities that appeared to participate in the replication of RNA could subsequently have turned out to be problematic or toxic. Thus, in the case of viral infections, the hypothesis contends that cellular pathology does not necessarily follow the entry of the virus, except as a symptom, as a visualization of the fact that the pathology is actually occurring. Instead, our hypothesis is that molecular structures, their activities, and the majority of the information that makes up this new pathological state does not follow the entry of the virus and is not a consequence of viral activity, but exactly the opposite; that latent pathology within the cell must be placed ahead of it, as the basis of the infection. In other words, it is what makes infection possible. The basis of the infection is already there because it is also the basis of the cell itself, “basis” in the sense of “origin”: this latent pathological cellular state would have been there all along since the beginning of the cell, and in its very development.

### Virus as Signal

To argue that the virus is a signal, or better, a folder full of signals, several conditions must be met. The first condition required for something to be considered a signal in a system is the existence of differentiated states which can communicate their difference through space and time (Yair 2011). The pathological state of the cell is different from the one we have characterized as the latent-pathological state of the cell (the healthy cell), and the virus seems to be the vehicle and the information (the signal) charged with transmitting this difference. We are moving from a dual model of virus infections (virus ↔ cell) to a triadic model (virus ↔ pathological state↔ transformation of the latent-pathological state into a pathological state) somewhat related to the Peirce’s triadic sign (signal ↔ object ↔ interpretant) (Marty 1990). In our hypothesis, the signal is the virus; the object, the origin and the cause of the virus, is the pathological state; and the interpretant is the transformation, which is induced by the virus, from a latent-pathological state (the apparently healthy cell capable of receiving the signal) into a new pathological state: is a self-transformation signaled by the virus.

The second condition which must be met is that the virus signal is subject to interpretation according to biochemical and molecular biology rules, through which the cell is informed that another molecular organization (i.e., the pathological state) is possible (semantic aspect). This interpretative aspect distinguishes the virus activity from that of a chemical process. The transmission of the viral information can be blocked in the initial phase of infection, or indeed at any point throughout the process. The viral process is not a chemical reaction determined from the outset. Moreover, many environmental and historical and contextual factors (i.e., temperature, history of previous infections, co-infections, cellular genetic instability, tissue cellular heterogeneity, etc.) intervene crucially in the development of viral signal interpretation. So, although the virus may transmit to healthy cells the information that another state is possible, this information must be contextually interpreted (pragmatic aspect) (Witzany 2006). In each new round of virus-cell entry, the interpretant results, eventually, in a very similar new pathological state (object 2) to that from which the virus signal derives (object 1), but it is not exactly the same. For RNA viruses two factors contribute to these subtle differences: firstly, each virus that infects a target cell is composed of a subset of genetic variants from a swarm of variants from the infected donor cell (del Portillo et al. 2011) (Jung et al. 2002); secondly, the cellular context: each cell might be subtly different, either for *in vitro* cell virus cultures (de la Torre et al. 1988) or for *in vivo* infections (Korber et al. 1994). Cells maintained in culture suffer from genetic instability, while an infected organism is essentially a mosaic of different environments (cells, cell types and physiological conditions), and the virus multiplication rate is strictly dependent on the environment (Domingo and Holland 1997). Moreover, in rare cases, the interpretant may lead to quite a different situation, for example, when a cell that is newly infected by a cytolytic virus does not develop a cytolytic infection but rather a persistent infection. Again, the reason for this could lie either with the virus (Labadie et al. 2004) or the host cells (de la Torre et al. 1985; Martin Hernandez et al. 1994). Other examples of drastic differences between object and interpretant are tropism changes promoted by changes of a virus’s use of cell receptors, and pathogenicity changes caused by substitutions in the viral polymerase and protease (Baranowski et al. 2001; Baranowski et al. 2003). Thus, virus signal meaning and evolution of meaning do not depend on the inherent properties of virus signal reproduction (i.e., high error rate) alone, but also in cellular contextual factors.

Thirdly, the grammatical rules, via which the RNA virus signal acquires meaning for those confronted elements within the cell, should be compatible with those we may presuppose to have applied to the RNA elements present at the origin of the cell. At least, high variation rates and limited message size, an inherent feature of RNA viruses, are accomplished (Martell et al. 1992; Domingo 2007). The analogy between language and the original RNA quasi-species structure (Eigen 1988), similar to that found in present-day RNA viruses, has already been put forward by Nowak (Nowak 2002).

We hypothesize that these pathological states are somehow equivalent to one of the cell’s ancient states. Each virally-dictated pathological state partially restores an early critical life situation. Viruses are signals derived from the biomolecules, activities and structures of life that existed in the remote past, but which are still present in our cells although in a fragmented, disjointed and inhibited form. In our hypothesis these pre-pathological states would be sustained by hidden agents, awaiting the arrival of an appropriate virus signal that tells them how to self-organize, express themselves, and so on.

## Discussion

### Evolutionary Forces That Might Maintain the Virus Signal

If viruses are signals, what selection forces could have acted to keep interpretation mechanisms within the cell that result, in many cases, in the death of that same cell? We come back to two selective forces, one is negative in character, and the other is positive. Negative selection forces would have preserved a risky feature or structure inside the cell in cases when, in addition to its potential toxic effects, it was also involved in activities that were essential to the cell. So instead of eliminating it, the cell somehow would have blocked its toxic properties. In fact, it is supposed that the most primitive molecular features are retained through selective pressure applied to their multiple connections and functions, which appeared as the complexity of cells increased in evolution. The presence of competing, at the time cooperating self-replicating agents and their swarms of associated biomolecules, might be sustained by their important contribution to fitness, rather than the pernicious effect of competition or mutual toxicity. Thus, the cell would have remained rooted in a state of tension, of latent internal conflict between its components, while it became more and more complex. This force would have retained the capability of the cell to interact/interpret the RNA virus signal since it first appeared. On the other hand, with regard to positive selection forces, we can conceive that in some cases, the cells having fortuitously incorporated stricter control mechanisms over their own internal elements would resist a new virus. In our hypothesis, this means that those cells would reaffirm cell unity when threatened with internal disintegration, generating the possibility for those new control systems to take over the metabolic networks affecting cell division and/or cell death, and/or acting to deal with situations of stress, and in any case conferring a competitive advantage over those cells which had never encountered that particular virus. In this sense, viral signals would favor cell complexity.

A particular virus signal that partially cuts off the communication between swarms of distinct cellular agents might lead to two different evolutionary effects: (i) in very primitive RNA-based cells more or less sensitive to the virus, the semiotic partitioning of a polymorphic population might lead to sympatric speciation; (ii) those cells that had developed immunity to the viral action would at least persist, and while contributing to the fitness of a polymorphic population they might subsequently be selected during a situation of stress or another type of infection. On the other hand, the high rates of RNA virus mutation may continuously introduce new challenges and lead to the selection of new solutions for the cells, for further application elsewhere and in future selection scenarios. Thus, sustained internal conflicts between cellular agents together with continuously arriving variants of virus signals create a trend towards increasing redundant cohesion mechanisms and cellular complexity. Lack of continuous viral infection (in the long term), would lead to the spontaneous atrophy of such redundant connections and interactions among cellular agents, weakening the cells and being eliminated via selection. It would therefore be possible to propose virus-cell relationships as an example of non-genetic, ecological inheritance (Danchin et al. 2011).

### Hidden Potential of RNA Signals

Studies in the 1970s indicated that rabbit reticulocyte ribosomes could bind to and protect authentic initiation regions of the bacterial phage f1 mRNA (Legon et al. 1977) and that eukaryotic viral RNAs, such as poliovirus, were translated into *E. coli* translation extracts (Rekosh et al. 1970). These studies indicated that at least a few signalling features within an mRNA strand, which can lead to ribosome recognition, should be common to prokaryotes and eukaryotes for initiation of protein synthesis (Legon et al. 1977). Recently, in the context of a non-canonical mechanism of translation initiation of Cricket Paralysis Virus (CrPV) (Dicistroviridae), Colussi (Colussi et al. 2015) found an RNA structural element capable of operating in both bacterial and eukaryotic translation systems, albeit with a different mechanism. It is likely that these events reflect neither the replication cycle of this virus in two such different hosts nor the canonical action of other bacteriophage mRNAs. Rather, it is more likely that the bacterial ribosome’s potential to bind the eukaryotic signal is reporting a very ancient type of interaction that has been preserved by other unknown means. These 5’ mRNA tRNA-like elements were found inside the viral mRNA as part of the internal ribosome entry site of the hepatitis C virus (Nadal et al. 2002) and then subsequently generalized to other viruses including animal pestivirus, picornaviruses and Cricket Paralysis Virus (CrPV) (Dicistroviridae) (Jan et al. 2003; Lyons and Robertson 2003). Very recently, we have identified and characterized structural elements bearing tRNA properties within human mRNA species (Díaz-Toledano and Gómez 2015) and in particular, in one of the characterized mRNAs, these structural elements coincide with what is known as a “cytoplasmic accumulation region”. Thus, what we find is: (1) the unexpected potential of a viral tRNA-like motif to retain “eventually” very ancient properties; (2) the resemblance structural RNA signals within cellular mRNAs have with tRNA. These may acquire the capacity to bind 40S ribosomal subunits or other unexpected activities after viral entry (for example, if the viral tRNA-like motif sponges out factors normally interacting with the tRNA-like-signals within cellular mRNAs, releasing them to carry out other activities); and (3) the ancient and central role of tRNAs in cell life. All together this could suggest the potential for a tRNA-like entity, or agent, capable of reproducing and representing (at least partially) a primordial form of early life in our present day cells.

### How to Support the Hypothesis Experimentally

The focus should be on identifying potentially hidden ontological entities within the cell at the molecular level by taking advantage of a viral infection. This involves, first, seeking out new structures and activities for ancient cellular RNAs that are acquired upon entry of the virus into the cell. Second comes the characterization of new interactions between these RNAs (e.g., ribosomal RNAs, or their degradation fragments after viral infection, RNA from RNase P; Signal Recognition Particle RNA, tRNAs, and other non-coding RNAs), nucleotides, cofactors and other cellular biomolecules that favor the multiplication of the virus. Third, it should be tested whether, after the entry of the virus, any of the cellular molecules identified rebel against the establishment of control over cellular unity. Then, there must be exploration of the relationships between these cellular molecules that both favor viral multiplication, and actively participate in disrupting the cellular *status quo.* The robustness of these “alliances” between similar viruses should be examined using examples from different genera. Fourth, the origins of these alliances should be traced back, if possible, to try to theoretically identify the ancient “beings” still living in our cells. The fifth and final step is to experimentally disrupt and reconstitute these beings (or parts of them), to prove their actual existence. If all this could be accomplished, a new antiviral strategy could be developed.

CIBERehd: Centro de Investigación Biomédica en Red de Enfermedades Hepáticas y Digestivas (ISCIII). CSIC: Consejo Superior de Investigaciones Científicas.
